# Genetic characterization of fall armyworm infesting South Africa and India indicate recent introduction from a common source population

**DOI:** 10.1371/journal.pone.0217755

**Published:** 2019-05-31

**Authors:** Rodney N. Nagoshi, Isabel Dhanani, R. Asokan, H. M. Mahadevaswamy, Chicknayakanahalli M. Kalleshwaraswamy, Sharanabasappa, Robert L. Meagher

**Affiliations:** 1 Center for Medical, Agricultural and Veterinary Entomology, United States Department of Agriculture-Agricultural Research Service, Gainesville, Florida, United States of America; 2 Plant Health Diagnostic Services, Department of Agriculture, Forestry and Fisheries (DAFF), Stellenbosch, South Africa; 3 Division of Biotechnology, Indian Institute of Horticultural Research (IIHR), Hessaraghatta Lake (PO), Bangalore, Karnataka, India; 4 Department of Entomology, University of Agriculture and Horticultural Sciences, Shivamogga, Karnataka, India; Northwest A&F University, CHINA

## Abstract

The invasion of the Western Hemisphere native fall armyworm (*Spodoptera frugiperda*; J. E. Smith) (Lepidoptera: Noctuidae) into the Eastern Hemisphere has been notable for the rapidity and geographical breadth of new detections. In the year following the first discovery in western sub-Saharan Africa in 2016, infestations have been documented in most sub-Saharan maize growing regions and has now expanded beyond Africa with populations recently reported in India. These observations could indicate a remarkable capacity for rapid establishment and long-distance dissemination. However, while fall armyworm does exhibit extended migration in North America where it annually traverses thousands of kilometers, this behavior is known to be dependent on highly favorable wind patterns and so can’t be assumed to occur in all locations. An alternative possibility is that the species has long been present in Africa, and perhaps the rest of the hemisphere, but was undetected until the enhanced monitoring that resulted after its initial discovery. Determining whether the fall armyworm in the Eastern Hemisphere is newly arrived or long pre-existing is important for assessing the risks of significant economic impacts, as the former indicates a change in pest composition while the latter does not. This study examined this issue by comparing collections from two geographically distant locations, South Africa and India. Sequence comparisons were used to quantify differences between the South Africa and India collections, assess the likelihood of their sharing a common source population, and their possible relationship with previously characterized fall armyworm from other regions of Africa. The results indicate genetic homogeneity between the South African and Indian fall armyworm populations tested and substantial similarities between these and collections from eastern Africa. The implications of these findings on fall armyworm population behavior and composition are discussed.

## Introduction

The fall armyworm, *Spodoptera frugiperda* (J. E. Smith) (Lepidoptera: Noctuidae), has long been a significant economic pest of maize and other crops in the Western Hemisphere [1]. It is a tropical species incapable of diapause and so has limited capacity to survive freezing winter temperatures, yet still has an infestation range that includes most of the maize-producing regions in North America [2]. This was shown to be due to long-distance migration behavior that allows northward dissemination over thousands of kilometers from wintering areas in southern Texas and Florida over the course of a single growing season [3, 4]. These long-distance movements are associated with, and likely dependent upon, air transport systems that show a strong and consistent northerly air flow across North America as well as a progressively northward expansion of maize agriculture during the spring-to-fall growing season that makes available a continuous food supply [5]. Therefore, while fall armyworm has the capacity for extensive migration, the extent and consistency of the behavior is dependent on environmental factors such that the behavior in North America may not be the norm.

The observations of fall armyworm in the Eastern Hemisphere is noteworthy for the geographical breadth of confirmed infestations that occurred shortly after the first detection. Within two years of the initial report of fall armyworm in Western Africa in 2016 [6], the species has been found in most sub-Saharan nations ranging from Kenya to the east and South Africa to the south [7–11], with recent confirmations of establishment at multiple locations in India [12–15]. There is considerable uncertainty as to what this pattern of detections is revealing about fall armyworm population behavior with two broadly defined alternative scenarios possible, 1) a recent successful introduction followed by rapid and long-distance dissemination, or 2) a long-time and pervasive but until recently unidentified fall armyworm presence in Africa (and perhaps other parts of the Eastern Hemisphere). In the first case, the spate of recent detections would be an accurate reflection of population movements while the second results from the enhanced monitoring resulting from the initial discovery.

Distinguishing between these scenarios is important for assessments of the risks and consequences of fall armyworm infestations. If fall armyworm is a recent introduction into the Eastern Hemisphere, then it represents a significant change in pest composition and the worst-case scenarios of additional agricultural losses are plausible. Alternatively, if the pest has long been present but undetected, then its recent discovery represents no change in the status quo and therefore should have no new economic consequences. In addition, estimating the frequency and locations of successful introductions from the Western Hemisphere are important to risk assessments, in particular the likelihood that deleterious resistance traits characterized in the Americas will make its way into the Eastern Hemisphere fall armyworm populations.

One strategy for addressing these issues is to use genetic comparisons to determine the relatedness of geographically separated populations. The first regional survey of a mitochondrial (*Cytochrome oxidase I* or *COI*) and a nuclear (*Triosephosphate isomerase* gene or *Tpi*) marker found only a small number of haplotypes that were common to all collection sites in western, central, and eastern Africa [9, 10]. This observation of low genetic variation suggests a small number of introductions that occurred too recently for new alleles to accumulate, while the genetic homogeneity is consistent with high mobility such that the geographically distant regional populations are derived from a common source or are frequently and extensively mixing.

Complicating the description of fall armyworm is the subdivision of the species into two “host strains” that differ in their distribution among certain host plants in the field. The “rice-strain” is preferentially found in pasture grass and millet while the “corn-strain” predominates in maize and sorghum [16, 17]. This host plant bias shows a consistent but not absolute correspondence with genetic polymorphisms in the *COI* and *Tpi* genes that themselves are usually but not always in agreement [18–20]. This occasional inconsistency between the markers and host plants combined with the morphological similarity between strains (they are effectively indistinguishable) leads to some uncertainty in strain identity. But the correspondence is sufficient to show that marker-defined strains exhibit differences in female pheromone constitution [21–23], mating behavior [24], and mating compatibility [25]. Multiple studies of Africa populations indicate that both strains are present based on the more commonly used *COI* marker, with the rice-strain predominating [6, 7, 9–11, 26]. In contrast, the *Tpi* marker shows the same collections to be almost entirely (>95%) of the corn-strain [9, 10]. This level of disagreement between the markers is rare in Western Hemisphere collections but is consistently observed in surveys throughout Africa, making the strain composition of the African populations uncertain. It is therefore not clear whether the current African fall armyworm population will have the same broad host range (>80 host species) as that documented for Western Hemisphere fall armyworm.

In this study we assessed the relationship between fall armyworm populations from two geographically distant locations separated by approximately 7,000 km. Specimens were obtained during 2017–2018 from maize fields in central and southern regions in South Africa, representing the most southern locations so far tested in the Eastern Hemisphere, and these were compared to collections from multiple locations and host plants in the state of Karnataka, India. Four gene segments were analyzed by DNA sequencing, three of which were previously used in the analysis of northern sub-Saharan populations [9]. These include the segment of the *COI* gene typically used for DNA barcoding [27] (COIA), a downstream segment of *COI* with polymorphisms that identify region-specific fall armyworm populations in the Western Hemisphere [28–30] (COIB), and a portion of the *Tpi* coding region (TpiE4) with strain-diagnostic polymorphisms [19, 20]. The fourth genetic segment is a portion of a *Tpi* intron that was previously shown to be highly polymorphic in Florida fall armyworm populations [31]. These sequence comparisons were used to quantify differences between the South Africa and India collections, assess the likelihood of their sharing a common source population, and their possible relationship with previously characterized fall armyworm from other regions of Africa.

## Results

### Species identity

**Fig 1:**
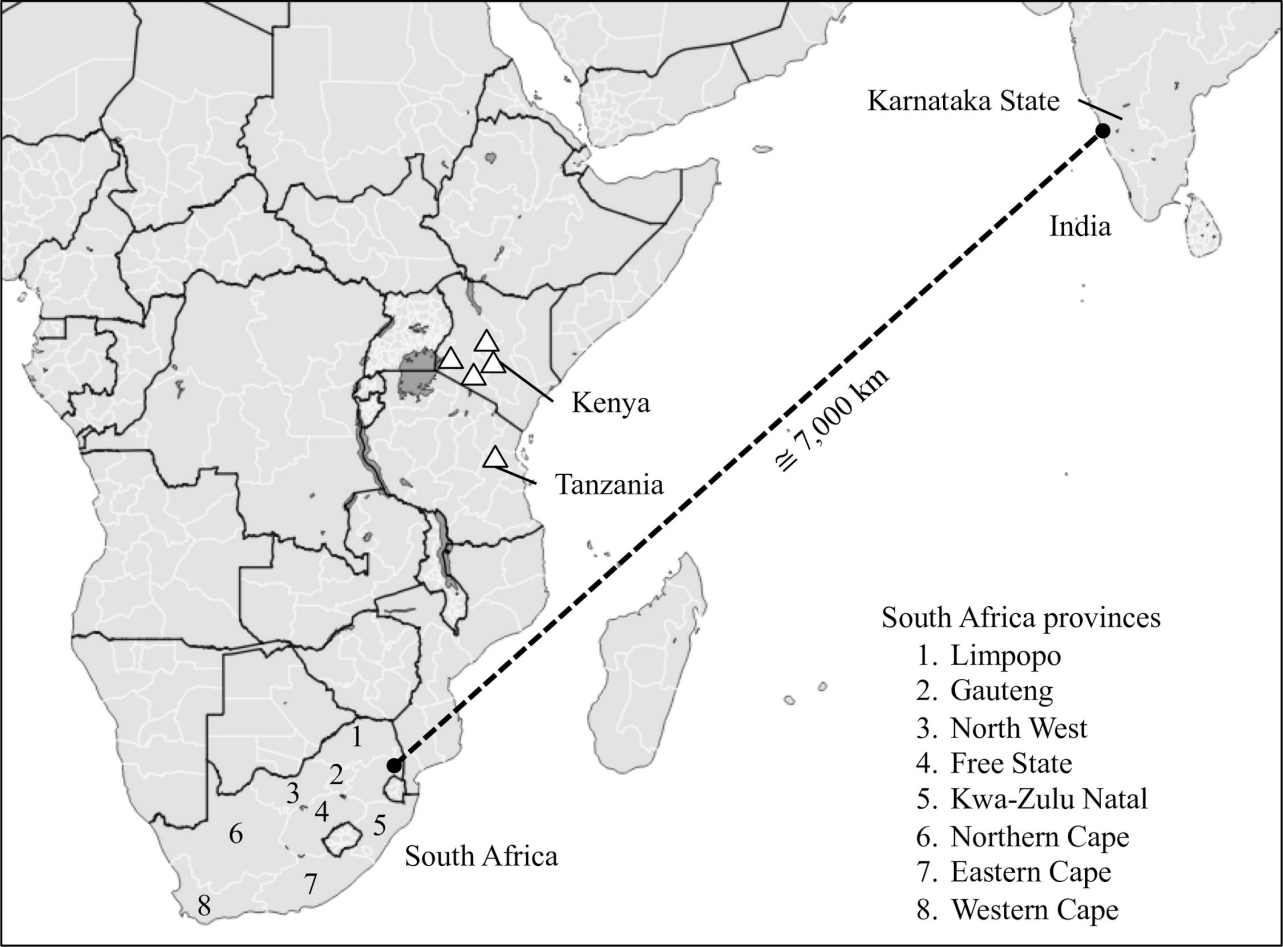
Map showing locations of fall armyworm collections from Africa and India. South Africa fall armyworm were from the eight provinces listed by number. India fall armyworm were collected from sites in the state of Karnataka, which is approximately 7,000 km distant from South Africa. Triangles indicate locations of fall armyworm previously sampled for genetic markers and discussed in this study [9].

**Fig 2:**
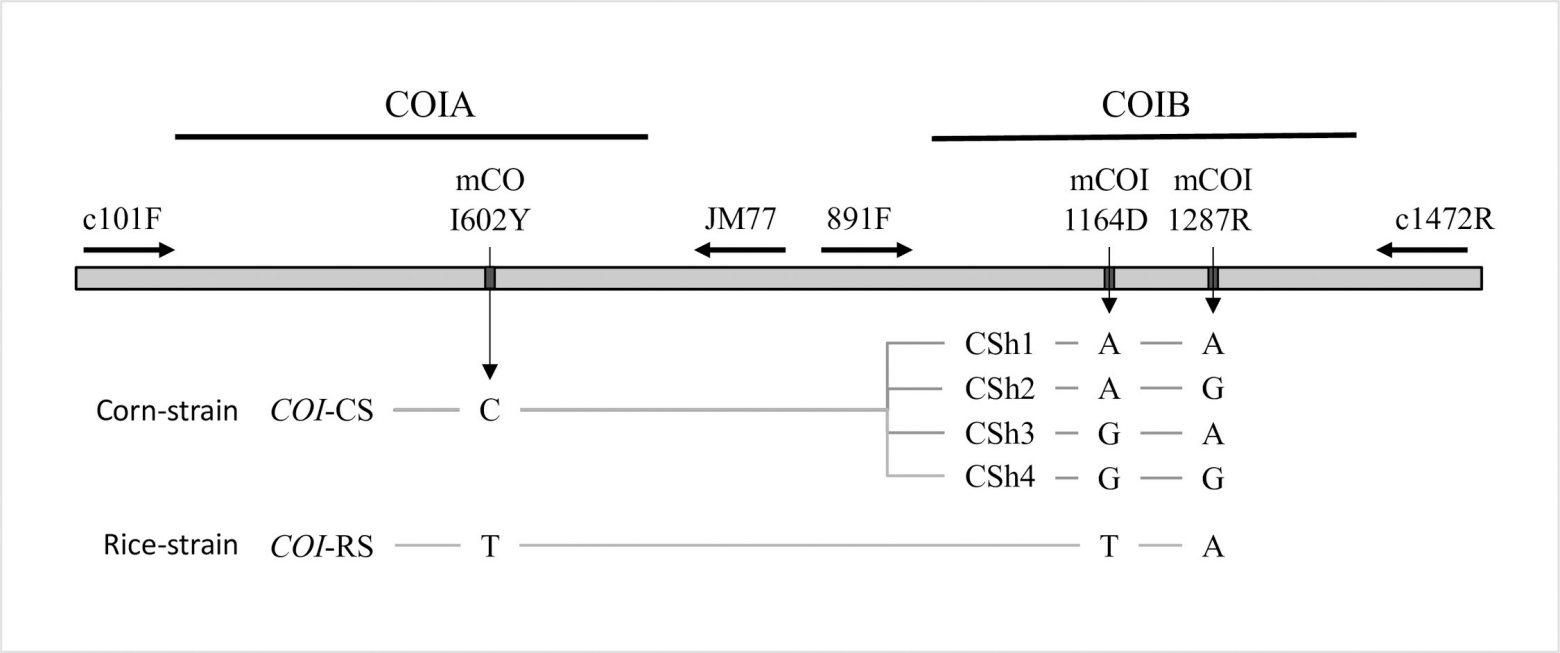
DNA segments from the *COI* gene showing polymorphic sites diagnostic for strain identity or that define important haplotypes. The locations of primers used for PCR amplification and DNA sequencing are provided (←, →) as are the locations of the COIA and COIB segments (—). The loci mCOI602Y and mCOI1164D identify strain-diagnostic sites with the polymorphic alternatives and their strain or haplotype definitions listed below. Site mCOI1287R is polymorphic but not strain-specific.

Moth larvae and adults were recently collected from locations in India and South Africa (Fig 1, Table 1). On site identification as fall armyworm was based on morphological characteristics and, in the case of adults, attraction to a pheromone blend designed for fall armyworm. Genetic characterization of the *COI* barcode region (COIA, Fig 2) identified one specimen as *Mythimna* (*Leucania*) *loreyi* (Duponchel), which was not further characterized. The Indian and South African specimens were composed of the same two COIA haplotypes at nearly the same proportions. The majority haplotype was identical in the sequenced segment to the most common corn-strain fall armyworm sequence found in the Western Hemisphere [27] (CS01, HM136586), which was present in 94% (44/47) and 93% (26/28) of the South Africa and India specimens tested, respectively. In both countries the remainder was made up of the most common rice-strain Western Hemisphere haplotype, RS09 (HM136601). In comparison, Florida fall armyworm analyzed for segment COIA found 14 sequence variants, five of the *COI-*CS type and nine in the *COI-*RS category [27].

**Table 1:** Source information for fall armyworm larval (L) and pheromone trap (T) collections.

Country	State or Province	Year	Host	Type	n
South Africa	Limpopo	2017	Maize	L	3
South Africa	Limpopo	2018	Maize	T	20
South Africa	Gauteng	2018	Maize	L	4
South Africa	Gauteng	2018	Maize	T	4
South Africa	North West	2018	Maize	L	2
South Africa	Free State	2018	Maize	L	1
South Africa	Kwa-Zulu Natal	2018	Maize	T	3
South Africa	Northern Cape	2018	Maize	T	3
South Africa	Eastern Cape	2017	Maize	L	1
South Africa	Eastern Cape	2018	Maize	T	5
South Africa	Western Cape	2018	Maize	T	3
India	Karnataka	2018	Rice	L	9
India	Karnataka	2018	Maize	L	19
From past studies.					
Kenya [9]	Multiple locations	2017	Maize	L	55
Tanzania [9]	Multiple locations	2017	Maize	L	69
United States [19, 32]	Florida	2002–8	Maize, Turf	L	104
Brazil [33]	Mato Grosso	2005	Maize, Cotton, Pasture, Rice, Millet	L	81

### Strain identity

**Fig 3:**
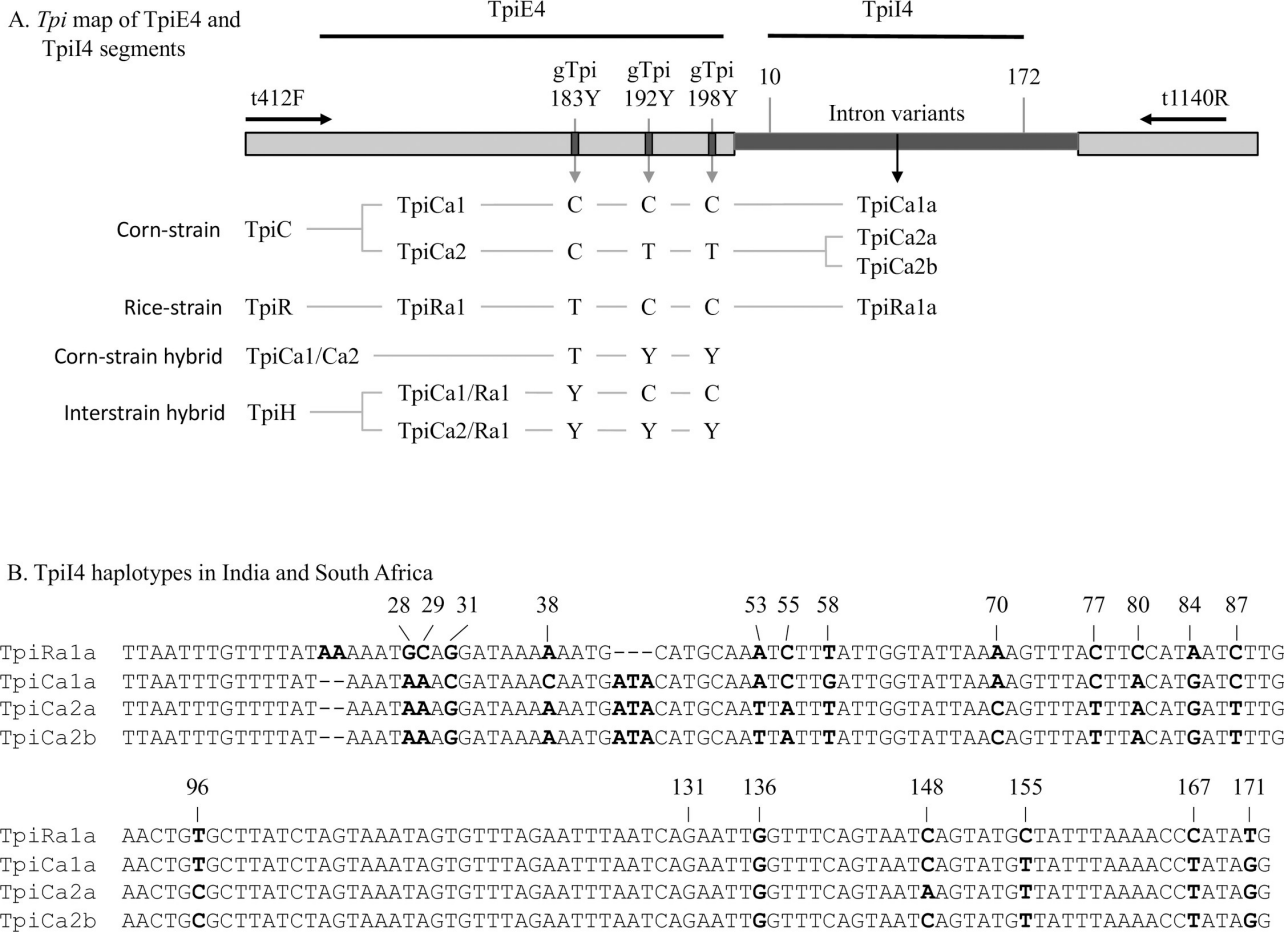
*Tpi* gene segments showing polymorphic sites diagnostic for strain identity or that define important haplotypes. The locations of primers used for PCR amplification and DNA sequencing are provided (←, →) as are the locations of the TpiE4 and TpiI4 segments (—). (A) The gTpi183Y site is diagnostic for TpiC and TpiR strain designations as well as TpiH heterozygotes, while polymorphisms at gTpi192Y and gTpi198Y identify within strain variants. The TpiI4 segment is defined by sites 10 and 172 within the intron. (B) Sequences of the four TpiI4 haplotypes found in India and South Africa collections. Letters in dark bold identify observed sites of polymorphism in these collections. Site 131 was associated with consistently poor sequence quality and so was assumed to be the consensus G for all specimens.

**Fig 4:**
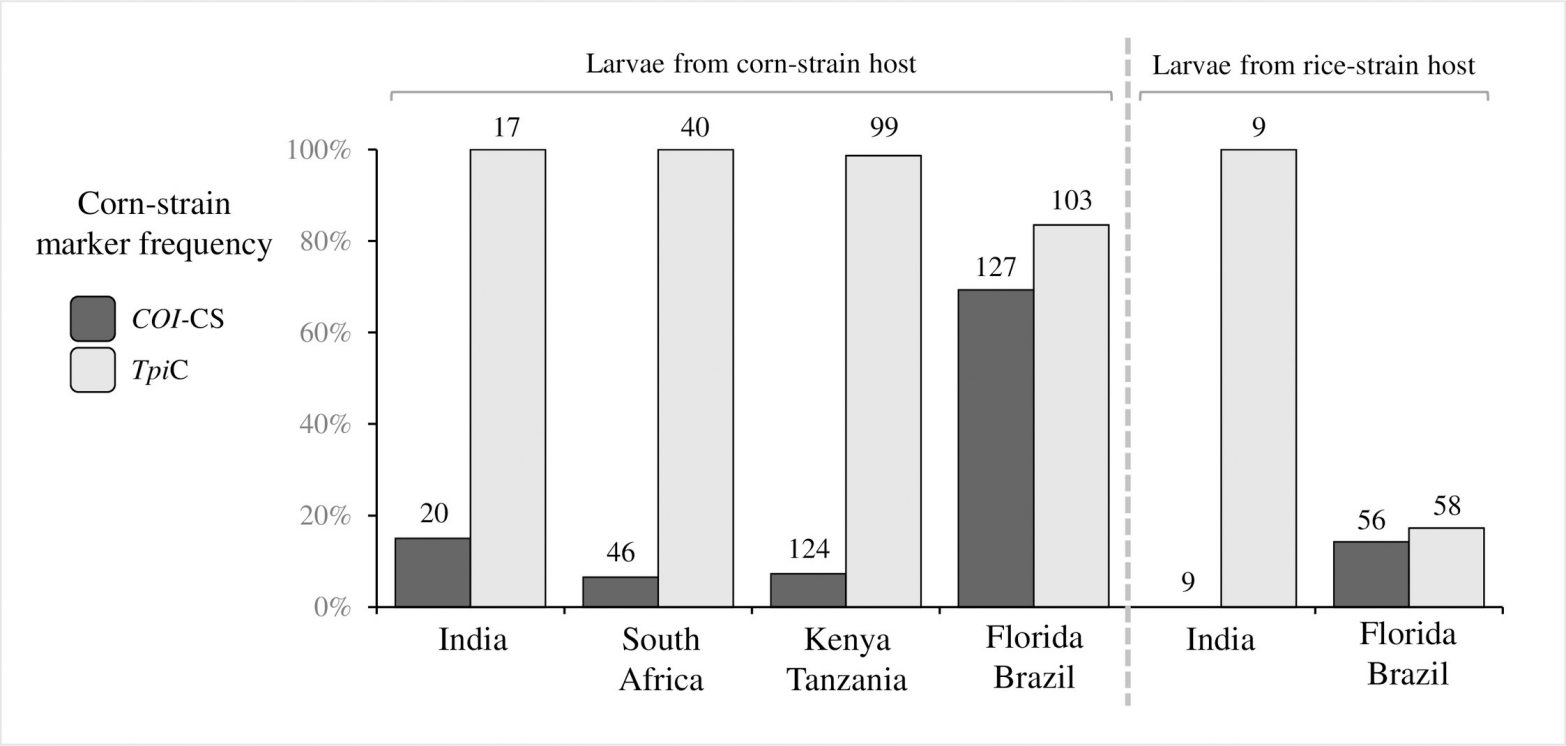
Frequencies of *COI-*CS and TpiC specimens in India, Africa, and the Western Hemisphere. *COI-*CS identity is defined by COIA or COIB polymorphisms, TpiC by the gTpi183A or gTpi183G polymorphisms. Data from Kenya-Tanzania and Florida-Brazil from previous studies (Table 1). Corn-strain hosts include maize and sorghum. Rice-strain hosts include rice, turf/pasture grasses, millet. Numbers above columns indicate number of total samples tested.

Strain identity was determined by examination of strain-biased polymorphisms in the COIB and TpiE4 gene segments (Figs 2 and 3A). The two markers are independent (one mitochondrial and the other nuclear) and in the Western Hemisphere displays a consistent, but not absolute, biased distribution on different host plants. In general, agreement between host plant and either the *COI* or *Tpi* markers is approximately 70% in corn-strain hosts and 90% in rice-strain preferred plants [18, 19]. A different profile is consistently observed in the Eastern Hemisphere. Collections from Indian and South African maize fields did show the expected predominance of the TpiC marker but were also primarily *COI*-RS, with only 4% of the South Africa and 15% of the India specimens displaying the expected *COI-*CS marker (Fig 4). This profile is similar to that found in Kenya and Tanzania in an earlier survey of northern sub-Saharan fall armyworm populations [9]. Only a small number of specimens from a single collection were available from a rice-strain host as these have to date been difficult to find in the Eastern Hemisphere. The collection from rice in India was all *COI*-RS and TpiC, similar to what was found in nearby maize fields (Fig 4).

### COIB haplotype comparisons

**Fig 5:**
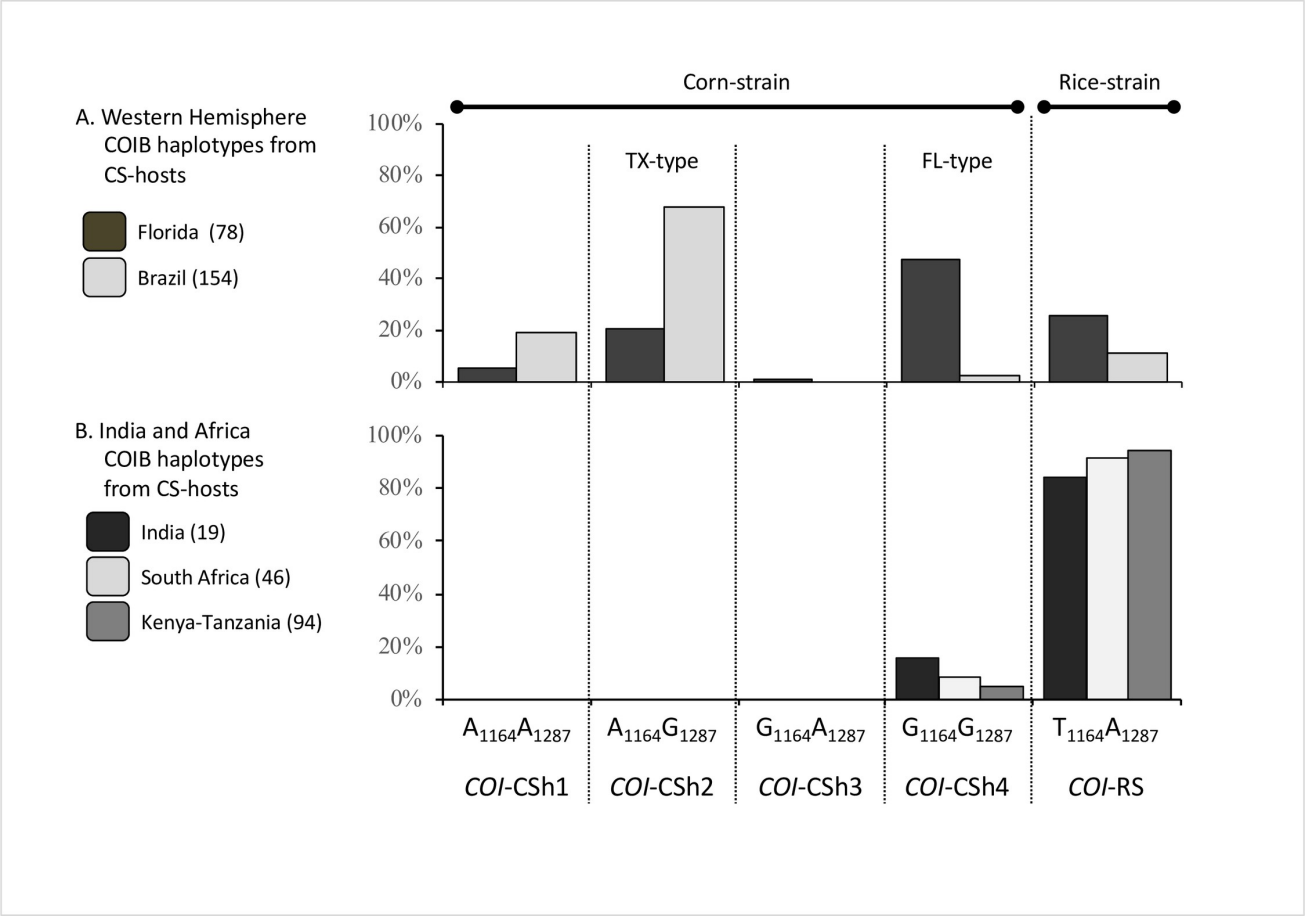
Frequencies of the COIB strain and CSh haplotypes that identify regional subpopulations in the Western Hemisphere. The CSh haplotypes are defined by polymorphisms at mCOI1164D and mCOI1287R. Collections are from maize or sorghum fields only. Numbers in parentheses indicate the samples tested. (A) Collections from Florida are representative of the FL-type population while those from Brazil have the profile of the TX-type. (B) Results from collections from maize fields and plants in India and South Africa compared to a previous study of fall armyworms from Kenya and Tanzania [9].

The COIB segment contains the polymorphic mCOI1164D and mCOI1287R sites that together produce five haplotypes, one indicative of the rice-strain (T_1164_A_1287_) and four (CSh1-4) that are *COI-*CS variants based on their linkage with the COIA strain markers (Fig 2). The CSh1-4 haplotypes can be found throughout the Western Hemisphere but differ in their relative proportions depending on host plant and location [3, 30]. The *COI-*CSh2 haplotype predominates in Texas, Brazil, and the rest of South America (called the TX-type) while the *COI-*CSh4 variant is the majority form found in populations that winter in Florida (FL-type, Fig 5A). The rice-strain T_1164_A_1287_ combination is typically in the minority in collections from maize.

In comparison, the T_1164_A_1287_ configuration makes up 90% or more of the fall armyworm tested in India and South Africa, a result consistent with that obtained from the COIA analysis (Fig 5B). The minority *COI-*CS types (six specimens) in both countries were all *COI-*CSh4, indicative of the FL-type. These results are similar to that observed for collections from Kenya and Tanzania [9] (pooled data Fig 5B).

### *Tpi* haplotype comparisons

The fourth exon of the presumptive *Tpi* coding region (TpiE4) contains several polymorphic sites in addition to the strain diagnostic gTpi183Y locus [19] (Fig 3A). In a previous study corn-strain fall armyworm from several locations in Africa were found to consist of three TpiE4 variants, one in the rice-strain TpiR category (TpiRa1, MH729873) that was found in <1% of the specimens, and two corn-strain TpiC haplotypes where TpiCa1 (MG603702) was the predominant (mean 83%) form at all locations followed by TpiCa2 (MG603703).

The India and South Africa collections are comprised of identical haplotypes at similar frequencies. The TpiCa1 haplotype predominates in both countries, making up 81% of the Indian and 90% of the South African collections, with TpiCa2 filling in the rest. Evidence for the presence of the TpiRa1 haplotype comes from one Indian specimen and five from South Africa that have sequence chromatographs consistent with heterozygosity between TpiRa1 and either TpiCa1 or TpiCa2 (TpiH, Fig 3A). The data suggest that TpiRa1 is sufficiently infrequent that homozygosity or hemizygosity for the allele is rare. A similar result was observed in an earlier survey of Africa that did not include South Africa (or India) where 12% of the tested specimens were TpiH compared to only 1% that were TpiRa1[9].

**Fig 6:**
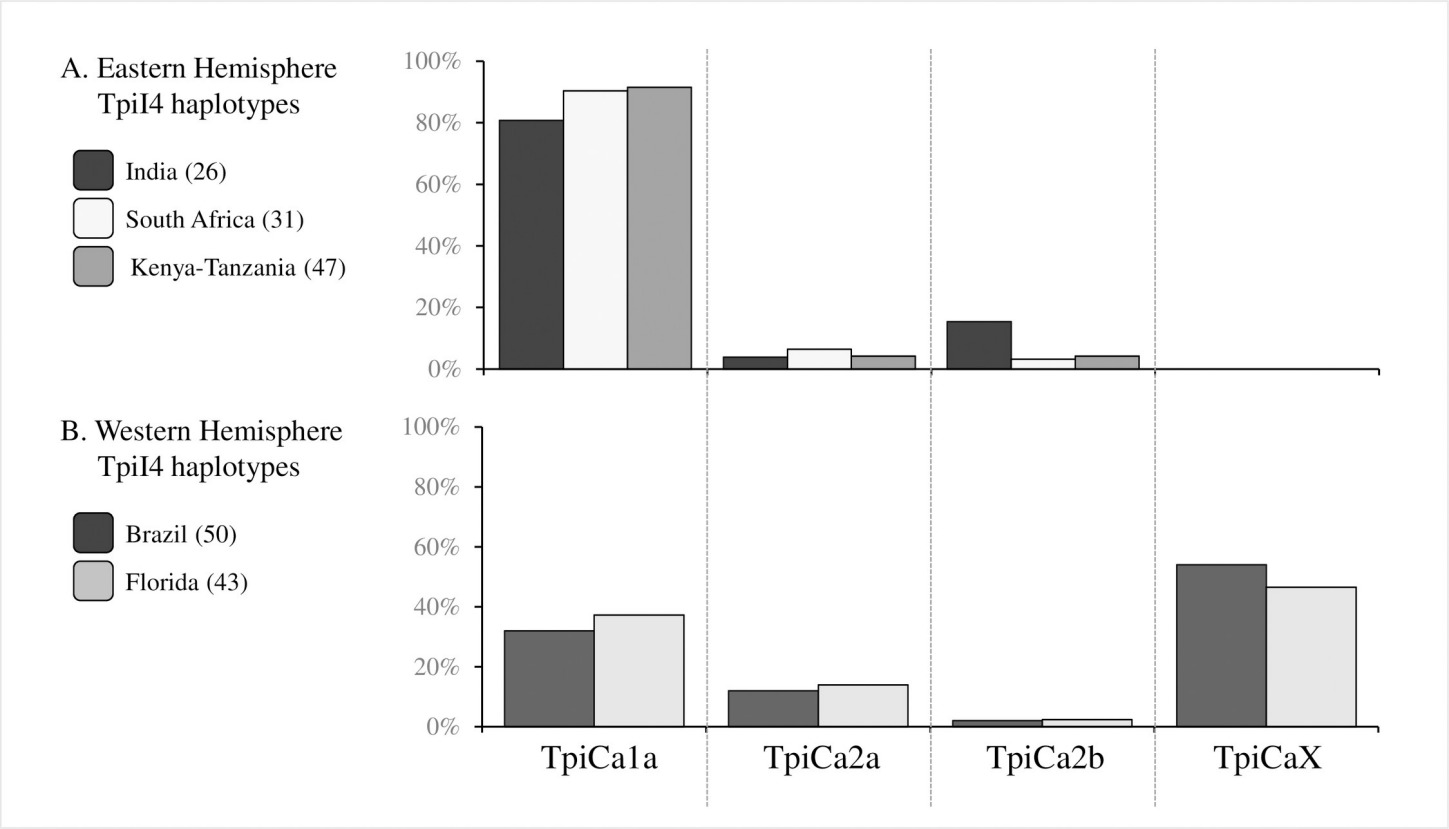
Frequencies of sequence variants in the TpiI4 segment. Number of specimens tested in parentheses. (A) TpiI4 haplotype frequencies from the Eastern Hemisphere are predominantly TpiCa1a, with low frequencies of TpiCa2a and TpiCa2b. (B) TpiI4 haplotype frequencies from fall armyworms captured in Florida and Brazil.

The *Tpi* introns displayed higher genetic variation than *COI* or *Tpi* exon regions and so in principle should be more likely to identify differences between populations [31]. Comparisons of the TpiI4 intron segment (Fig 3B) showed that the collections from India and South Africa had similar compositions both to each other and to fall armyworm from Kenya and Tanzania (Fig 6A). Each were composed of the same intron variants in roughly the same proportions, with the majority represented by TpiCa1a (MH726218), which is a variant of TpiE4 haplotype TpiCa1, and minor amounts of TpiCa2a (MH726219) and TpiCa2b (MH726220), both associated with exon haplotype TpiCa2 (Fig 3B). This pattern differs from that seen in the Western Hemisphere, where about 60% of the specimens tested were of intron variants other than TpiCa1a, including about 40% with sequences not found at this time in Africa (TpiCaX, Fig 6B).

### Genetic variation comparisons

The collections from India, South Africa, and pooled samples from Kenya and Tanzania were analyzed for genetic variation metrics for the COIB and TpiI4 segments and compared to populations from the Western Hemisphere (Brazil and Florida, Table 2). Overall, the collections from India and Africa had lower haplotype diversity, nucleotide diversity, and theta/site values than those from the Western Hemisphere. Statistically significant differences were found for haplotype diversity and theta/site with COIB and for all three metrics with TpiI4. The variability metrics were typically lower with COIB compared to TpiI4, consistent with variation in the former being constrained by being a protein coding sequence.

**Table 2:** Descriptive statistics of polymorphisms found in the COIB and TpiI4 segments in collections from different locations (host plant of collected larvae, CS = corn-strain, RS = rice-strain).

			Haplotype diversity	Nucleotide diversity	
Collection	n	Haplotypes	Hd ± SD	pi ± SD	Theta/site
A. COIB					
India (CS, RS)	28	2	0.20 ± 0.09	0.006 ± 0.003	0.007
South Africa (CS)	46	2	0.16 ± 0.07	0.005 ± 0.002	0.006
Kenya-Tanzania (CS)	102	3	0.15 ± 0.05	0.003 ± 0.001	0.005
Brazil (CS)	136	26	0.75 ± 0.04	0.008 ± 0.001	0.015
Florida (CS)	48	13	0.80 ± 0.04	0.015 ± 0.006	0.010
B. TpiI4					
India (CS, RS)	26	3	0.34 ± 0.11	0.020 ± 0.006	0.016
South Africa (CS)	31	3	0.19 ± 0.09	0.011 ± 0.005	0.015
Kenya-Tanzania (CS)	51	3	0.19 ± 0.07	0.010 ± 0.004	0.013
Brazil (CS)	41	15	0.88 ± 0.04	0.039 ± 0.005	0.047
Florida (CS)	56	18	0.75 ± 0.06	0.056 ± 0.008	0.073
C. Two-tailed *t*-test: (India, South Africa, Kenya-Tanzania) vs. (Brazil, Florida)					
Metric	DNA	*P*	*t*	*df*	*r*^*2*^
Haplotype diversity	COIB	0.0002*	22.30	3	0.99
Nucleotide diversity	COIB	0.0958	2.40	3	0.66
Theta/site	COIB	0.0479*	3.24	3	0.78
Haplotype diversity	TpiI4	0.0057*	7.12	3	0.94
Nucleotide diversity	TpiI4	0.0207*	4.48	3	0.87
Theta/site	TpiI4	0.0188*	4.65	3	0.88

SD, Standard deviation.

*Significant to *P* < 0.05

## Discussion

There are multiple insect species where recurring seasonal migration over thousands of kilometers have been documented [34–36]. Most relevant is evidence that the Globe Skimmer dragonfly undergoes seasonal migrations between India and eastern Africa, which includes a trans-oceanic crossing of 3500 km that most likely uses the high-altitude winds associated with the Inter-tropical Convergence Zone [35]. This provides a mechanism for how fall armyworm might have arrived in India as well as the possibility of regular interactions between the Indian and African fall armyworm populations through natural migration. However, while fall armyworm is documented to undergo annual long-distance migration in North America [37], the plausibility of an analogous journey between Africa and India is uncertain. Fall armyworm has only been observed to undergo continuous flight from dusk to dawn (6–12 hours) using regional air transport systems [5]. This typically limits a single flight to a few hundred kilometers, making traversing the 3500 km crossing of the Arabian Sea seemingly problematic given known fall armyworm behavior.

The genetic homogeneity observed between the South Africa and India collections with respect to the small number of *COI* and *Tpi* haplotypes strongly suggests that each arose from a common source. The fall armyworm from both nations displayed the same *COI* haplotype class that predominates in the rest of Africa and that we previously reported most likely originated from Florida or the Caribbean [9, 10]. The lack of genetic variation was observed even with the highly polymorphic *Tpi* intron segment that previous studies showed had a nuclear diversity (pi) value in Western Hemisphere populations that was 3- fold higher than that of the adjacent exon segment [31]. Despite this variability, only three intron haplotypes were detected and these were found in both South Africa and India.

Perhaps most consistent with a recent common origin is the similarity in the relative frequencies of the different haplotypes. The COIA, COIB, TpiE4, and TpiI4 haplotypes displayed nearly identical frequency profiles in the South Africa and India collections. In particular, collections from both countries showed the same disagreement between the diagnostic *COI* and *Tpi* strain markers, where the large majority of specimens displayed the mitochondrial rice-strain marker *COI-*RS (>80%) and also the nuclear corn-strain marker TpiC (>90%). These markers are typically in agreement in the Western Hemisphere fall armyworm collections, making it unlikely that the presence of the same anomalous haplotype profile in both India and South Africa could be coincidence. Disagreement between *COI* and *Tpi*-based strain identification could occur as a result of hybridization between strains [19], and there is evidence for such an event in the presumptive fall armyworm propagule that established the Africa population [38].

It will be informative to determine if this extensive genetic similarity between the South African and Indian fall armyworm populations persists over time. If the populations are reproductively isolated then a gradual divergence will be observed both in haplotypes and their relative frequencies, while continued homogeneity would suggest substantial interactions despite the long geographical distances and physical barriers present between locations.

The genetic results to date are most consistent with a small number of successful introductions (perhaps only one) from the Western Hemisphere that subsequently dispersed to form the current fall armyworm infestations in the surveyed locations in South Africa and India. In particular, the results in this paper are consistent with a previous survey of fall armyworm populations from the northern portion of sub-Saharan Africa that also compared the COIA, COIB, and TpiE4 segments, but not the TpiI4 intron segment [9]. The South Africa and India collections showed nearly identical haplotype profiles with fall armyworm from Kenya and Tanzania, suggesting a close relationship and recent interactions between these populations. We note that eastern Africa lies between India and South Africa (by land) and that fall armyworm was reported in Kenya and Tanzania before South Africa and India. If the temporal sequence of detections is an accurate indicator of first arrival, then it appears that eastern sub-Saharan Africa is the likely source of the populations sampled by the India and South Africa collections.

Whether these conclusions apply more generally is unclear, particularly for India. Only two locations and two plant hosts from a single state in India were surveyed with relatively small numbers from each, so the data may not be representative of the total Indian fall armyworm population. It is possible that as sampling increases additional *COI* and *Tpi* variants will be found that may indicate a different origin. This may be the case for the *COI* segments as there are reports of higher genetic variability in the COIA segment in other India fall armyworm collections than we observed [15]. More extensive surveys with both the *COI* and *Tpi* markers are clearly needed.

Despite these caveats, we believe the results to date indicate substantial homogeneity in the genetic composition of the fall armyworm found in India and South Africa, as well as to other surveyed populations in Africa, most notably Kenya and Tanzania. The evidence favors a common and recent origin for the fall armyworm infesting India and South Africa that at its simplest would involve a single successful introduction followed by long distance dissemination to India and South Africa through natural or trade-assisted migration.

## Materials and methods

### Collections

Fall armyworm were collected as larvae from maize and rice plants in the state of Karnataka, India (Fig 1, Table 1), and processed for DNA isolation. An aliquot of DNA for each specimen was sent to CMAVE (Gainesville, FL) for subsequent analysis. The South Africa collections were from pheromone traps (adult males) placed in or near maize fields and larvae (gender unknown) collected from maize plants. Adult males were air dried after collection. Collected larvae were placed in 70%-90% ethanol. Both were stored at ambient temperature. Specimens were processed for DNA isolation at CMAVE (Gainesville, FL). No endangered or protected species were involved in this study. We obtained permission from private farmers for access and data collection in their fields.

### DNA preparation and PCR amplification

India collections were processed at the Indian Council of Agricultural Research-Indian Institute of Agricultural Research (ICAR-IIHR) [15]. The larval specimens were rinsed with double distilled water to remove residual ethanol, then homogenized in 500 μl CTAB extraction buffer (100 mM Tris-HCl pH 8.0, 1.4 M NaCl, 0.02 mM ethylene diamine tetraacetic acid, 2X CTAB, 2X PVP and 2 μl αβ-mercaptoethanol). The homogenized mixture was incubated for 30 min at 65°C with occasional mixing by tube inversion. An equal volume of chloroform-isoamyl alcohol (24:1) was added, mixed, and the sample was centrifuged for 10 min at 10,000 rpm at 4°C. The final aqueous phase obtained was transferred into a new tube; an equivalent volume of ice-cold isopropanol was added. The tubes were centrifuged at 13000 rpm for 15 min. The pellet was dried and dissolved in RNase/DNase-free water (30μl). Finally, 1 ml RNase (1 mg/ml) was added to each tube for RNA removal and then incubated for 1 h at 37°C. The DNA solution was stored at -20°C until further use.

South Africa adult specimens were dissected with a scalpel with either the head or abdomen used for DNA isolation and the remainder stored in 100% ethanol at -20°C. Larval segments of approximately the same mass were used for DNA analysis with remainders (if any) also archived in ethanol. Each tissue fragment was placed in a 2-ml threaded microcentrifuge tube with 7 stainless steel beads (2.8 mm, OPS Diagnostics, Lebanon, NJ) and 0.5 ml of cell lysis buffer (100 mM Tris-HCL, 150 mM NaCl, 50 mM EDTA, 0.5% SDS, pH 7–8). Tissues were homogenized using the Mini-BeadBeater 96 (BioSpec Products, Bartlesville, OK) for 30 seconds as per manufacturer’s instructions. After homogenization, the tubes were cooled on ice. Another 0.5 ml of cell lysis buffer was added, followed by incubation at 55°C for 10 minutes. Samples were centrifuged at 12000 rpm for 10 minutes. The supernatant was collected and placed in Zymo-Spin III columns (Zymo Research, Orange, CA) and processed according to manufacturer’s instructions. The DNA preparation was increased to a final volume of 80 μl with distilled water. DNA preparations were stored at -20°C.

PCR amplification for all segments was performed in a 30-μl reaction mix containing 3 μl 10X manufacturer’s reaction buffer, 1 μl 10mM dNTP, 0.5 μl 20-μM primer mix, 1 μl DNA template (between 0.05–0.5 μg), 0.5 unit Taq DNA polymerase (New England Biolabs, Beverly, MA). The thermocycling program was 94°C (1 min), followed by 33 cycles of 92°C (30 s), 56°C (45 s), 72°C (45 s), and a final segment of 72°C for 3 min. Typically 96 PCR amplifications were performed at the same time using either 0.2-ml tube strips or 96 well microtiter plates. All primers were obtained from Integrated DNA Technologies (Coralville, IA). Amplification of the *CO1* barcode region (COIA) was performed using primers *COI-**101F* (5’- TTCGAGCTGAATTAGGAACTC -3’) and *COI-678R* (5’- ATAGGATCACCTCCTCCTGCA-3’) to produce a 569-bp fragment (Fig 1). Amplification of the *CO1* segment used to determine region-specific haplotypes (COIB) used the primer pair *891F* (5’-TACACGAGCATATTTTACATC-3’) and *1472R* (5’-GCTGGTGGTAAATTTTGATATC-3’) to produce a 603-bp fragment. Amplification of the *Tpi* gene segment containing both TpiE4 and TpiI4 used the primers *412F* (5’- CCGGACTGAAGGTTATCGCTTG -3’) and *1140R* (5’- GCGGAAGCATTCGCTGACAACC-3’).

For fragment isolations, 6 μl of 6X gel loading buffer was added to each amplification reaction and the entire sample run on a 1.8% agarose horizontal gel containing GelRed (Biotium, Hayward, CA) in 0.5X Tris-borate buffer (TBE, 45 mM Tris base, 45 mM boric acid, 1 mM EDTA pH 8.0). Fragments were visualized on a long-wave UV light box and manually cut out from the gel. Fragment isolation was performed using Zymo-Spin I columns (Zymo Research, Orange, CA) according to manufacturer’s instructions. The University of Florida Interdisciplinary Center for Biotechnology (Gainesville, FL) and Genewiz (South Plainfield, NJ) performed the DNA sequencing.

### Characterization of the *CO1* and *Tpi* gene segments

The genetic markers are all single nucleotide substitutions. Sites in the *COI* gene are designated by an "m" (mitochondria) while *Tpi* sites are designated "g" (genomic). This is followed by the gene name, number of base pairs from the predicted translational start site (*COI*) or 5' start of exon (*Tpi*), and the nucleotides commonly observed using IUPAC convention (R: A or G, Y: C or T, W: A or T, K: G or T, S: C or G, D: A or G or T).

The *COI* markers are from the maternally inherited mitochondrial genome. Two adjacent segments of *CO1* were analyzed by DNA sequencing. The COIA segment was used to confirm species identity and to determine the fall armyworm *COI* strain haplotypes *COI-*CS (C-strain) and *CO1*-RS (R-strain). The COIB segment was amplified by *CO1* primers 891F and 1472R and used to confirm host strain identity and determine the region-specific haplotypes (Fig 2A). The DNA sequences of the fall armyworm host strains and other *Spodoptera* species were previously described and available in GenBank [27].

Variants in the *Tpi* gene can also be used to identify host strain identity with results generally comparable with the *CO1* marker [19, 20]. The gTpi183Y site is in the fourth exon of the predicted *Tpi* coding region and was PCR amplified using the *Tpi* primers 412F and 1140R (TpiE4, Fig 3). The C-strain allele (*Tpi*C) is indicated by a C_183_ and the R-strain (*Tpi*R) by T_183_ [10]. The *Tpi* gene is located on the *Z* sex chromosome that is present in one copy in females and two copies in males. Since males can be heterozygous for *Tpi*, there is the potential for the simultaneous display of both alternative nucleotides at *Tpi*_183_ (denoted as *Tpi*H), which would be indicated by an overlapping C and T DNA sequence chromatograph [39].

The TpiI4 intron segment was sequenced used primers 412F for the initial sequencing reaction and 1140R for 2^nd^ strand sequence confirmation when needed in cases of ambiguity. Intron length is variable in the Western Hemisphere because of frequent insertions and deletions (indels) [31]. Sequence comparisons were made with a 162 bp segment beginning 10 bp from the intron 5’ start (Fig 3A). This segment was chosen for analysis because it empirically had the most consistent sequence quality with the given primers and had fewer indels in Western Hemisphere specimens than other intron segments, both factors that simplified the sequence comparisons. One site, gTpI4[131]R, was problematic in that it consistently gave poor signal and high background. Given this ambiguity, the consensus nucleotide (G_128_) was assumed (Fig 3B).

### Data analysis

DNA alignments and consensus building were performed using MUSCLE (multiple sequence comparison by log-expectation), a public domain multiple alignment software incorporated into the Geneious Pro 10.1.2 program (Biomatters, New Zealand, http://www.geneious.com) [40]. Phylogenetic trees were graphically displayed in a neighbor-joining (NJ) tree analysis also included in the Geneious Pro 10.1.2 program [41]. The adjacent COIB segment was used to confirm host strain identity and determine the region-specific haplotypes (Fig 1) [3, 30]. Descriptive DNA sequence statistics and calculations of nucleotide variation based on the Jukes-Cantor model [42] were performed using DNAsp Ver. 6.12 [43]. Generation of graphs were done using Excel and Powerpoint (Microsoft, Redmond, WA). Statistical analyses including *t*-tests were performed using GraphPad Prism version 7.0 for Mac (GraphPad Software, La Jolla California USA).
